# Genetic Dissection of Phomopsis Stem Canker Resistance in Cultivated Sunflower Using High Density SNP Linkage Map

**DOI:** 10.3390/ijms21041497

**Published:** 2020-02-22

**Authors:** Zahirul I. Talukder, William Underwood, Guojia Ma, Gerald J. Seiler, Christopher G. Misar, Xiwen Cai, Lili Qi

**Affiliations:** 1 Department of Plant Sciences, North Dakota State University, Fargo, ND 58108, USA; zahirul.talukder@usda.gov (Z.I.T.); guojia.ma@ndsu.edu (G.M.); xiwen.cai@ndsu.edu (X.C.); 2 USDA-Agricultural Research Service, Edward T. Schafer Agricultural Research Center, 1616 Albrecht Blvd. N., Fargo, ND 58102-2765, USA; william.underwood@usda.gov (W.U.); Gerald.Seiler@usda.gov (G.J.S.); christopher.misar@usda.gov (C.G.M.)

**Keywords:** sunflower, Phomopsis stem canker, disease resistance, quantitative trait loci (QTL), genotyping-by-sequencing, single nucleotide polymorphism (SNP) markers

## Abstract

Phomopsis stem canker (PSC) caused by *Diaporthe helianthi* is increasingly becoming a global threat for sunflower production. In this study, the genetic basis of PSC resistance was investigated in a recombinant inbred line (RIL) population developed from a cross between HA 89 (susceptible) and HA-R3 (resistant). The RIL population was evaluated for PSC disease incidence (DI) in seven screening trials at multiple locations during 2016–2018. The distribution of PSC DI in the RIL population was continuous, confirming a polygenic inheritance of the trait. A moderately high broad-sense heritability (*H*^2^, 0.76) was estimated for the trait across environments. In the combined analysis, both the genotype and the genotype × environment interactions were highly significant. A linkage map spanning 1505.33 cM was constructed using genotyping-by-sequencing derived markers. Marker–trait association analysis identified a total of 15 quantitative trait loci (QTL) associated with PSC resistance on 11 sunflower chromosomes, each explaining between 5.24 and 17.39% of the phenotypic variation. PSC resistance QTL were detected in two genomic regions each on chromosomes 3, 5, 13, and 17, while one QTL each was detected in the remaining seven chromosomes. Tightly linked single nucleotide polymorphism (SNP) markers flanking the PSC resistance QTL will facilitate marker-assisted selection in PSC resistance sunflower breeding.

## 1. Introduction

Phomopsis stem canker (PSC) caused by the Ascomycete fungus *Diaporthe helianthi* Munt.-Cvet., Mihaljc. & M. Petrov (anamorph *Phomopsis helianthi*) is endemic to sunflower production areas worldwide [1]. Severe outbreaks of PSC can lead to early senescence, plant wilting/lodging, or stem breakage [2], resulting in yield losses of up to 40% [3] and loss of oil content up to 25% [4]. The disease was first observed in the former Yugoslavia in the late 1970s [5]. Although, the disease was first reported in the USA in 1980, it has not been considered a serious threat to USA sunflower production until recently. A gradual increase of PSC severity has been observed in the USA since 2005 [6,7]. However, a dramatic increase in the prevalence of PSC disease in the USA Northern Great Plains has been reported since 2009 in the annual sunflower crop survey coordinated by the National Sunflower Association [7,8,9,10,11]. Significant damage to sunflower crops was also reported in 2009 in parts of Australia, especially in areas with extended wet periods [12,13], indicating that PSC is becoming a global threat to sunflower production.

Host resistance has been considered one of the most effective tools for disease management. The outbreak of PSC in Yugoslavia in the early 1980s led to an intensive search for resistance in existing inbred lines and commercial hybrids. Mihaljčević et al. [14] reported significant differences in PSC disease severity among hybrids and inbred lines in field trials where inbred lines originating from crosses of *Helianthus annuus* × *H. tuberosus* consistently showed the greatest ability to survive. Cuk [15] reported that wild *H. debilis* and *H. pauciflorus* were free of *D. helianthi* infection and are potential sources of resistance to PSC. Škorić [16] summarized the results of field screening trials conducted over a five-year period, including over 7000 sunflower inbred lines, experimental hybrids, and varieties and found only four lines with a high degree of tolerance to PSC.

Field screening of United States Department of Agriculture (USDA) sunflower germplasm resources for resistance to PSC was initiated in the early 1990s with resistance identified in multiple USDA plant introduction (PI) lines [17,18,19,20]. The upward trend of PCS disease prevalence since 2009 in USA sunflower fields has led to an intensive search for new sources of resistance in existing USDA inbred lines and PI collections. Gulya et al. [21] reported screening of 1106 PIs at three locations under natural infection and found 23 (2.08%) PIs showing the highest levels of PSC resistance with <10% infection. Talukder et al. [22] screened a total of 227 PIs and 33 inbred lines for PSC resistance in 2011 and 2012 at multiple locations in North Dakota, South Dakota, and Minnesota, USA. Considerable genetic variation for PSC resistance was observed among the sunflower lines. The broad-sense heritability (*H*^2^) for the disease trait was 83%, sufficient to facilitate further PSC resistance breeding in sunflowers. A total of 36.9% of the lines showed significantly higher PSC resistance than the susceptible check (HA 89), and 13 PIs, mostly from Hungary, exhibited dual resistance against PSC and *Sclerotinia* head rot diseases. Mathew et al. [23] screened 49 sunflower accessions in the greenhouse using representative isolates of *D. helianthi*, the most common causal agent of PSC in the USA, and *D. gulyae*, another *Diaporthe* species causing PSC on sunflowers in the USA, and found that only one accession (PI 552939) was significantly less susceptible to both *Diaporthe* species than the susceptible check HA 288.

Initial genetic studies revealed that resistance to PSC was controlled predominantly by additive genetic effects with partial dominance [24]. Škorić [16] also found that resistance was controlled by partial dominance with at least two or more complementary genes. Tourvieille de Labrouhe et al. [25] indicated that resistance is partly under the control of recessive genes but also depends on the interaction between a number of genes. In a later study, Vrânceanu et al. [26] confirmed that the hybrids of a diallel cross showed resistance that was under predominantly additive control but with some partial dominance and proposed the hypothesis that only a small number of genes control resistance. However, in subsequent years, when a larger number of observations were available, it became evident that a continuous range of reactions existed among sunflower populations, from extremely susceptible to highly resistant, which suggested that PSC resistance is quantitative in nature and governed by mostly additive gene action [27,28,29,30,31,32].

The lack of sunflower germplasm resources exhibiting complete resistance to PSC along with the complex, quantitative nature of resistance to this disease presents a substantial challenge for breeding efforts to introduce PSC resistance into elite sunflower lines with valuable agronomic traits. Moreover, multiple *Diaporthe* species have been reported to cause PSC in sunflowers [10,13,33,34,35] further complicating efforts to improve resistance through breeding. A detailed investigation conducted in Australia reported that the pathogenic *Diaporthe* isolates associated with the PSC outbreak are different from those found in both Europe and the USA [13,33]. The causal agents were determined to be of three novel *Diaporthe* species, a highly damaging *D. gulyae* sp. nov. and two less damaging *D. kochmanii* sp. nov. and *D. kongii* sp. nov. species. In the USA, *D. helianthi* and *D. gulyae* were identified as the two predominant *Diaporthe* spp. causing PSC in surveys conducted from 2010 to 2012 in the Northern Great Plains, with *D. helianthi* more commonly associated with PSC than *D. gulyae* in this study [10,11].

Currently, there is little information about the genetic loci controlling PSC resistance in sunflowers. Only two reports have been published describing quantitative trait loci (QTL) mapping of PSC resistance in sunflowers [36,37]. Bert et al. [36] detected a total of 15 QTL across several linkage groups (LGs) of the sunflower genome using an F_2_-derived F_3_ population. Those QTL explained from 7.2 to 34.7% of the phenotypic variation for PSC resistance. Langar et al. [37] identified PSC resistance QTL on eight chromosomal regions in a recombinant inbred line (RIL) population. A major QTL was detected on LG15 that explained 46% of the phenotypic variation for frequency of attack at flowering under semi-natural infection. These studies used AFLP (amplified fragment length polymorphism), RFLP (restriction fragment length polymorphism), and DALP (direct amplification of length polymorphisms) DNA markers for linkage mapping. The laborious nature of evaluating these markers makes them unsuitable for breeding applications, and they have been superseded by single nucleotide polymorphism (SNP) markers as the preferred marker system for use in breeding programs. Consequently, additional studies are needed to identify PSC resistance QTL using high-throughput SNP markers that can be used in marker-assisted selection (MAS) breeding.

The objective of the current study was to investigate the genetic basis of PSC resistance in sunflowers using a recombinant inbred line population developed by crossing two inbred lines exhibiting contrasting levels of PSC resistance and genotyped with high-throughput SNP markers. A total of 15 QTL associated with PSC resistance were identified, and tightly linked SNP markers flanking the PSC resistance genes/QTL were reported, which will facilitate MAS breeding. 

## 2. Results

### 2.1. Phomopsis Disease Screening of Sunflower RIL Population

**Figure 1:**
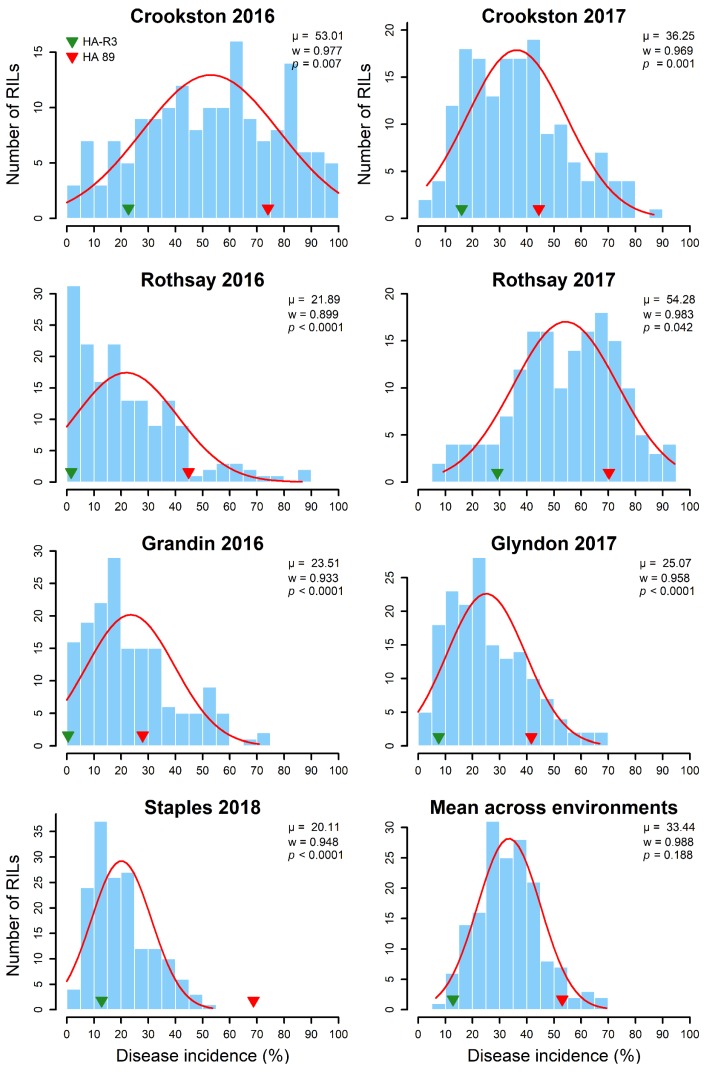
Frequency distribution of Phomopsis stem canker disease incidence (DI) among 164 sunflower recombinant inbred lines tested in multiple environments during 2016 to 2018. The arrowheads indicate the DI levels of the parental lines, HA-R3 (green) and HA 89 (red). The Shapiro–Wilk normality test statistic (*w*), the probability value (*p*), and the mean (*µ*) of the data for each environment are shown inside the respective plots.

Prevalence of PSC disease was observed in all seven environments (locations and years) where the parents and the HA 89/HA-R3 RIL population were evaluated. The highest PSC incidence was observed in the Rothsay 2017 environment (mean DI, 54.3%), followed by Crookston 2016 (mean DI, 53.0%) and Crookston 2017 (mean DI, 36.3%), suggesting that the climatic conditions were more conducive for PSC disease development in Crookston in both 2016 and 2017 and Rothsay in 2017 (Figure 1 and Figure S1). The remaining four environments had relatively lower PSC DI but were comparable, ranging from 20.1 to 25.1%. The mean PSC DI across all seven environments in the RIL population was 33.4%. Continuous distributions of PSC DI scores were observed within the RILs in all seven environments, consistent with quantitative disease resistance (Figure 1). The parental lines of the mapping population showed clear separation with respect to PSC DI in all environments with mean DI of 12.8 and 53.1% for HA-R3 and HA 89, respectively. In all seven environments, some of the RILs showed more extreme phenotypes than either of the parents, suggesting transgressive segregation of the trait where both the parents of the RIL population are contributing to the PSC disease resistance (Figure 1).

A Shapiro–Wilk normality test [38] revealed that the distributions of PSC DI data were not normal for any of the individual environments, but the combined means across all environments were normally distributed. The distributions of PSC DI data at Rothsay 2017 and Crookston 2016 were largely skewed toward the higher DI values. In contrast, the distributions of PSC DI data of the remaining five environments were skewed toward the lower values. The widest PSC DI range was observed in the Crookston 2016 environment (0 to 100%), followed by the Rothsay 2017 environment (9 to 94%), while it was the lowest in the Staples 2018 environment with 0 to 54% (Figure 1). The remaining four environments had similar PSC DI ranges (0 to 87%).

Analysis of variances (ANOVA) revealed highly significant genetic variation (*p* < 0.001) for PSC DI in the RIL population in all seven environments. In the combined analysis, both the genotypes, and the genotype × environment interactions were highly significant, suggesting contributions of both genetic and environmental factors in the observed phenotypic variation of PSC DI scores (Table 1). A moderately high broad-sense heritability (*H*^2^, 0.76) was estimated for the PSC trait on an entry mean basis across all the environments. The Spearman rank correlations (ρ) between PSC DI scores in the sunflower RILs tested in multiple environments of North Dakota and Minnesota are presented in Table 2. Significant positive correlations of various magnitudes were observed among all environments, except between the Crookston 2016 environment and the Rothsay 2017 and Glyndon 2017 environments.

### 2.2. Linkage Map Construction

Final linkage analysis was performed using 1879 SNP markers in the HA 89/HA-R3 RIL population with 1393 unique loci mapped to 17 sunflower chromosomes corresponding to 17 linkage groups (LGs) (Table 3). Out of the 1879 SNPs, 928 SNPs were obtained from variants calling against the HA412.v2.0 sunflower reference genome sequence, while the remaining 951 SNPs were obtained from calling against the HanXRQr1.0 reference genome sequence (Table S1). A total of 276 (14.7%) markers were distorted (*p* < 0.05) from the expected 1:1 segregation ratio. The highest number of distorted markers were mapped in LG17 (51 SNP), followed by LG2 (43 SNP) and LG13 (28 SNP), while the lowest number of distorted markers was mapped in LG15 (2 SNP) (Table S2). Among 1879 mapped SNPs, 340 SNPs were mapped to other LGs conflicting with their original linkage designation (Tables S1 and S2). A total of 486 (25.9%) SNP markers co-segregated and were mapped with other SNPs in the linkage groups. The highest number of co-segregating SNPs were mapped in LG8 (60), while the lowest number were mapped in LGs 5, 6, and 12 (14 SNPs each). The total length of the genetic map spanned 1505.34 cM with an average of one marker in 0.80 cM and one locus in 1.08 cM distance across the sunflower genome. The length of individual LGs ranged from 40.43 (LG6) to 133.38 cM (LG16), while the number of markers ranged from 60 (LG7) to 206 (LG8) (Table 3).

### 2.3. Quantitative Trait Loci Analysis

**Figure 2:**
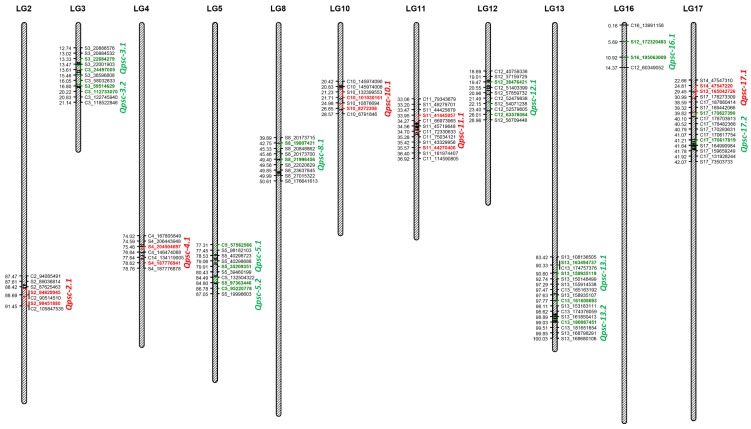
Quantitative trait loci (QTL) associated with Phomopsis stem canker (PSC) resistance identified in the HA 89/HA-R3 recombinant inbred line population tested in multiple environments during 2016 to 2018. The alleles for increased PSC resistance contributed by the HA-R3 parent are indicated in green, while the resistance alleles contributed by the HA 89 parent are indicated in red. The colored SNPs indicate the markers flanking PSC resistance QTL.

Combined QTL analysis identified a total of 15 QTL associated with PSC resistance on 11 sunflower chromosomes, each explaining between 5.24 and 17.39% of the phenotypic variation (PV) (Table 4, Figure 2). Ten of these genomic regions had positive QTL alleles that reduced the PSC DI derived from the resistant parent HA-R3, while the remaining five genomic regions had positive alleles derived from the susceptible parent HA 89. PSC resistance QTL were detected in two genomic regions each on LGs 3, 5, 13, and 17, while only one genomic region was detected with PSC resistance QTL in each of the remaining seven LGs 2, 4, 8, 10, 11, 12, and 16 (Figure 2). The highest number of individual environment QTL were detected in the *Qpsc-8.1*, *Qpsc-13.2*, and *Qpsc-17.2* QTL intervals on LGs 8, 13, and 17 with four QTL each (Table S3). All these QTL had positive alleles contributed by the resistant parent, HA-R3. In the *Qpsc-8.1* QTL interval, the four individual -environment QTL were detected in Crookston, Grandin, and Rothsay in 2016 and the Staples 2018 environments within a 5.3 cM genomic region, each explaining between 7.03 and 14.48% PV, and the additive effects ranged between 2.77 to 7.83 for the trait in a single environment (Table S3). At the lower end of LG13, the four individual-environment QTL were detected in Crookston, Glyndon, and Rothsay in 2017 and Crookston 2016 environments within a 4.5 cM region of the *Qpsc-13.2* QTL, each explaining between 5.24 to 11.0% of the PV and the additive effects ranged between 4.05 to 6.14. In the *Qpsc-17.2* QTL interval, another four individual-environment QTL were detected within a short span of the 1.0 cM genomic region in Grandin and Rothsay in 2016 and the Crookston 2017 and Staples 2018 environments, each explaining between 5.04 and 17.87% of the PV of the PSC DI with additive effects ranging from 3.82 to 9.35.

Three individual-environment QTL each were mapped on genomic intervals of *Qpsc-5.1* and *Qpsc-5.2*, both located on LG5 with PSC-resistant QTL alleles derived from the resistant parent, HA-R3 (Table S3). In the 2.2 cM genomic region of *Qpsc-5.1*, three individual-environment QTL were detected in the Grandin 2016 and Glyndon and Rothsay 2017 environments, each explaining between 5.37 and 7.69% of PV with additive effects ranging between 3.62 and 7.69. Another three individual-environment QTL in the *Qpsc-5.2* region were detected in Grandin and Rothsay in 2016, and Crookston in 2017, each explaining between 5.88 and 6.74% of PV, and with additive effects ranging from 4.23 to 4.49. The QTL on LG11, *Qpsc-11.1* also had three individual-environment QTL mapped within a 4.2 cM genomic region where the PSC-resistant QTL alleles were derived from the susceptible parent, HA 89 (Table S3). Each of these individual-environment QTL explained between 8.49 and 14.00% of PV with additive effects ranging between 2.94 and 7.37. Single individual-environment QTL were mapped to genomic regions *Qpsc-2.1* and *Qpsc-3.2*, while two individual-environment QTL were mapped in each of the genomic regions for the remaining seven QTL, *Qpsc-3.1*, *Qpsc-4.1*, *Qpsc-10.1*, *Qpsc-12.1*, *Qpsc-13.1*, *Qpsc-16.1* and *Qpsc-17.1* (Table S3).

## 3. Discussion

Phomopsis stem canker has been considered a yield-limiting factor for sunflower production in Europe since it was first reported in the former Yugoslavia [5]. The prevalence of PSC disease on sunflowers in the cool and humid Northern Great Plains has been increasing over the past two decades [7]. Changing climatic conditions with increased precipitation and a warmer and longer growing season in the North Central States, USA, have likely played a critical role in the PSC dynamics in this region [39]. The recent surge of PSC disease on sunflowers in the USA necessitates research to develop disease management options and improved disease resistance in order to maintain the competitiveness of the crop. PSC disease management using fungicide treatment has not been promising due to the difficulties associated with the number and appropriate timing of fungicide application [39]. The use of host resistance is the most efficient and economic option to combat the disease. Initial searches for sources of PSC disease resistance revealed that considerable variation in resistance exists within the USDA sunflower collections [22]. To gain further insight into the genetics of PSC resistance, a mapping population consisting of 164 RILs was developed from the cross of two sunflower inbred lines, HA 89 and HA-R3, each with a contrasting response to the PSC disease. In the absence of a suitable artificial inoculation method for large-scale field screening trials, we relied on natural PSC infection and evaluated the RIL population in seven environments (location × year) throughout North Dakota and Minnesota, USA, during the 2016 to 2018 growing seasons. 

PSC screening locations were carefully chosen based on the reports of recurring prevalence of the disease to ensure the availability of natural inoculum. All the screening environments in the current study showed moderate to high levels of PSC DI (Figure 1). The frequency distribution of PSC DI in all the environments was continuous, ranging from highly resistant to highly susceptible reactions, as expected for a quantitatively inherited trait. Some of the RILs even showed a more extreme phenotype than either of the parents in all the screening trials, suggesting transgressive segregation of the trait where both the parents contributed to the expression of the phenotype. In addition to the genetic variation, a substantial influence of the environment was observed in the form of significant G×E interaction for the trait in the RIL population (Table 1). Phomopsis disease is favored under conditions of abundant moisture with optimum temperature ranging between 23 and 25 °C during the growing period [1]. Variable agro-climatic factors across the screening environments (location and year) coupled with prevailing *Diaporthe* pathogen populations might have contributed to the significant G×E interaction.

Recent investigations identified multiple *Diaporthe* species in the Northern Great Plains area capable of causing PSC on sunflowers with *D. helianthi* more prevalent than *D. gulyae* [11]. While both fungal species are equally damaging to sunflowers, *D. gulyae* is more aggressive than *D. helianthi* with variable distribution of species across years and locations [11]. Currently, we do not have a comprehensive picture of the prevalence of the different *Diaporthe* species causing PSC at our different screening locations or across years. However, an initial investigation was undertaken to identify the species for a total of 13 samples collected from the nurseries in 2016 and 2017. From these samples, 12 were identified as *D. helianthi*, while only one was identified as *D. gulyae* (sample collected in Crookston in 2017). Whether pathotypes with distinct virulence profiles exist for any of the *Diaporthe* species causing PSC on sunflowers is also currently unknown. Despite significant G×E interaction, the Spearman rank correlations (ρ) among PSC DI scores of most of the environments were significant (Table 2), suggesting acceptable repeatability of the screening trials across different environments. The broad-sense heritability estimate of the trait was also moderately high (*H*^2^=0.76), as was observed in an earlier study [22], suggesting a reasonable prospect for genetic improvement of the trait through breeding.

We employed genotype-by-sequencing (GBS) [40] to simultaneously identify a large number of SNP markers and genotype the RIL population for linkage mapping. In the process of SNP calling, we took advantage of aligning the GBS sequence reads to the two independent sunflower reference genome assemblies, HA412.v2.0 [41] and XRQv1.0 [42] (https://www.heliagene.org/HanXRQ-SUNRISE). This approach helped us to achieve better coverage in the linkage map with respect to both marker resolution and absence of larger gaps. There were no gaps that exceeded 10 cM between loci in the current linkage map, while only 3.7% of the gaps were over 5 cM. The length of the current linkage map was 1505.33 cM, slightly longer than the previously reported sunflower linkage maps developed using SNP markers (1310.0 cM, [43]; 1443.84 cM, [44]; 1369.80 cM, [45]; and 1401.36 cM, [46]). The proportion of distorted SNP markers (14.7%) present in the current linkage map might have contributed to this length variation. Despite minor extension of the linkage map, inclusion of slightly distorted markers resulted in better marker grouping from the same chromosomes and increased genome coverage by markers and provided more information to the outputs from QTL mapping [47,48]. The flanking markers C13_161608693 and C13_180087451 of *Qpsc-13.2*, S14_47547220 and S13_165042726 of *Qpsc-17.1* and the left flanking marker S2_84829945 of *Qpsc-2.1* and S17_170827390 of *Qpsc-17.2* QTL were all distorted (*p* < 0.05) but provided valuable information regarding PSC resistance in the current mapping study.

QTL analyses were performed in each of the seven environments individually along with a combined analysis using best linear unbiased predictor (BLUP) extracted from PSC phenotypes across the seven environments. However, we reported only the 15 significant QTL that were detected in the combined analysis on eleven sunflower LGs, with the largest effect QTL mapped on LG17, explaining 17.39% of the PV in the mapping population (Table 4). Most of the QTL explained smaller PV for the PSC resistance, consistent with a quantitative trait that is controlled by many minor QTL. Individual environment QTL analysis also detected one to a maximum of four significant QTL within the genomic regions of the 15 QTL, although there were a few with minor discrepancies in peak QTL position (Table S3). The lack of congruency in the map position among the individual environment QTL might be attributed to the effects of different environments as observed in the significant G×E interaction for the trait.

Bert et al. [36] detected 15 PSC QTL on LGs 3, 4, 8, 10, 11, 14, and 17 of the sunflower genome. Except for LG14, PSC resistance QTL were detected on the same apparent linkage groups in the current study. Langar et al. [37] detected PSC resistance QTL on LGs 4, 6, 12, 13, 15, 16, and 18 in a RIL population derived from the cross of HA 89/LR4. Again, PSC resistance QTL were detected on LGs 4, 12, 13, and 16 in both this prior work and our current study. However, it is important to note that Bert et al. [36] reported a total of 19 LGs in their linkage map, whereas Langar et al. [37] reported 18 LGs in their linkage map. The number of LGs reported in these studies is higher than the number of diploid sunflower chromosomes (2n = 2x = 34). Moreover, these previous studies employed different DNA marker systems. Consequently, it is difficult to determine which of these LGs corresponds to which chromosomes in the sunflower genome. In contrast, the linkage map we present is comprised of 17 LGs, each corresponding to the 17 sunflower chromosomes and constructed using SNP markers, which have become the predominant DNA marker system in the modern genomics era [49]. Therefore, special care must be taken when comparing the results of the current study with the PSC resistance QTL published in the two earlier reports.

Bert et al. [36] reported two large-effect QTL on LGs 4 and 8, each explaining 34.7% of the PV for the trait. On the other hand, Langar et al. [37] reported a large-effect QTL on LG15, explaining 46% of the PV, although the authors presumed an overestimate of PV due to the larger map interval. Nonetheless, the QTL mapped in the current study were all small-effect QTL with the maximum 17.39% PV explained by a QTL on LG17 (Table 4). Overall, not only this study, but all the QTL mapping studies indicate that PSC resistance in sunflowers is a complex/polygenic trait controlled by several minor QTL, and many of those QTL are not always stable across environments. Therefore, marker-assisted selection for PSC resistance in sunflowers is likely to begin with a few loci exhibiting relatively large effects and consistent across environments [50].

Three RILs, RIL58, RIL82, and RIL101, presumably benefited from the transgressive segregation and outperformed the resistance parent, HA-R3, with lower PSC DI across the seven environments. Further genetic and field evaluations are underway with these three RILs as prospective candidates for future release.

## 4. Materials and Methods

### 4.1. Plant Materials

A RIL population consisting of 164 F_6_ progeny lines was developed by the single-seed descent method from a cross between sunflower inbred lines HA 89 (susceptible to PSC) and HA-R3 (resistant to PSC). HA 89 (PI 599773) is an oilseed maintainer line released by USDA-Agricultural Research Service (ARS) and the Texas Agricultural Experiment Station in 1971. HA-R3 (PI 650754) is a germplasm line developed and released by the USDA-ARS and the North Dakota Experimental Station in 1984 [51]. HA-R3 was originally released as a rust-resistant line of Argentine origin, which carries the rust resistance gene *R*_4_ [52,53]. Field screening trials at multiple locations of North Dakota, South Dakota, and Minnesota, USA, in 2011 and 2012 revealed that HA 89 was highly susceptible to PSC with a mean disease incidence (DI) of 39.8%, while HA-R3 was resistant with a mean DI of 8.4% [21,22].

### 4.2. Experimental Design and Phenotypic Evaluation

The RIL population along with the parents were evaluated for PSC resistance in field trials under conditions of natural infection at seven environments (years × locations) in North Dakota and Minnesota. Field trials were conducted at Grandin, ND, in 2016; Crookston and Rothsay, MN, in 2016 and 2017; Glyndon, MN, in 2017; and Staples, MN, in 2018. All field screening trials were conducted as a randomized complete block design (RCBD), each with three replications. Each plot consisted of a single 6 m row with a spacing of 75 cm between rows and thinned to approximately 25 plants per row.

Sunflower plants were evaluated for PSC DI at R9 growth stage (physiological maturity) [54]. Typical PSC symptoms develop at the node where the petiole meets the sunflower stem with a grey-to-dark brown necrotic lesion 15 to 20 cm in length centered around the petiole, which may girdle the stalk [1]. The fungus often damages the pith tissue beneath the lesion on susceptible genotypes, thus causing the stem to become hollow and prone to lodging. DI was expressed as the percent of plants showing PSC symptoms.

### 4.3. Statistical Analysis

ANOVA for PSC DI scores of the RIL population was performed individually for all seven environments using a generalized linear mixed model (Proc GLIMMIX) in SAS v9.4 [55]. Because our data represent the number of diseased plants out of the total number of plants grown in an experimental unit, a binomial distribution and a logit function were used for GLIMMIX procedure [56]. Variance components were estimated using Proc MIXED in SAS v9.4 (SAS, 2012) where all factors were treated as random effects. Broad-sense heritability (*H*^2^) was estimated on an entry mean basis following Nyquist [57]: $H^{2}=\sigma_{g}^{2}/\left(\sigma_{g}^{2}+\sigma_{ge}^{2}/l+\sigma_{e}^{2}/lr\right)$, where $\sigma_{g}^{2}$ is the genotypic variance, $\sigma_{ge}^{2}$ is the genotype × environment variance, $\sigma_{e}^{2}$ is the error variance, *r* is the number of replications, and *l* is the number of environments. Spearman’s rank correlation of PSC DI scores among trials was carried out using statistical package R v3.4.3 [58].

### 4.4. DNA Extraction and SNP Genotyping

The parents and the HA 89/HA-R3 RIL population were grown in the greenhouse in 36-well plastic trays, each containing nine rows of four 6.5 × 7.5 cm wells filled with ProMix BX potting media (Premier Horticulture Inc, Quakertown, PA). Leaf tissue from four young seedlings per RIL were bulked and freeze-dried. Genomic DNA was extracted from ~50 mg of leaf tissue per line using a Qiagen DNeasy 96 plant kit (Qiagen, Valencia, CA, USA) with a modified protocol described by Horne et al. [59]. The quality and the concentrations of the extracted DNA were measured using a NanoDrop 2000 Spectrophotometer (Thermo Fisher Scientific, Wilmington, DE, USA) and by electrophoresis running on a 1.5% agarose gel.

Approximately, 2 µg of high-quality genomic DNA from each of the 164 RILs and duplicate samples from two parental lines were sent to Data2Bio (Ames, IA) for GBS. The service company performed proprietary tunable GBS (tGBS^®^) technology that employs two restriction enzymes to generate overhangs in opposite orientations to which single-stranded oligos are ligated. This simplified GBS sequencing library preparation strategy ensures that only double-digested fragments are amplified and sequenced [60]. Sequencing was performed using four runs in an Ion Proton Instrument (Thermo Fisher Scientific, Wilmington, DE, USA), generating an average of 2,259,586 reads per sample. Prior to analysis of sequencing reads, the nucleotides of each read were scanned for low-quality bases, and those with PHRED quality <15 were removed. SNP identification was performed using a custom protocol in our lab. Briefly, the scanned reads were aligned to the two sunflower reference genomes, HA412.v2.0 [41] and HanXRQr1.0 (https://www.heliagene.org/HanXRQ-SUNRISE) using Bowtie2 aligner [61]. Putative variants were called using Freebayes [62] with initial filtering of sites for <20% missing data, >10% minor allele frequency, >20 QUAL score, and minimum read depth 3. The initial filtering retained 19,494 and 19,073 total variants, respectively, for the HA412.v2.0 and HanXRQr1.0 genome alignments. The final filtering was performed using the following criteria: (a) variants called consistently in both samples for each parent; (b) polymorphic between the two parents; and (c) called homozygous in both parents. The final filtering retained 3814 and 3800 good-quality variants (SNPs and indels), respectively, from the HA412.v2.0 and HanXRQr1.0 genome alignments for linkage mapping. The SNPs were named with a prefix of S1 to S17 (markers obtained from HA412.v2.0 genome alignment) and prefix C1 to C17 (markers obtained from HanXRQr1.0 genome alignment) based on the sunflower genome assemblies that correspond to the 17 sunflower chromosomes followed by a number representing the physical position of the SNP on the respective genome assembly. The sequences of SNPs associated with PSC-resistant QTL are presented in Table S4.

### 4.5. Linkage Mapping

The marker data were first analyzed in JoinMap 4.1 [63,64] to assess the goodness of fit to the expected 1:1 segregation ratio of the RIL population using the Chi-square test. To reduce the calculation burden, we used the “similarity loci” feature of JoinMap and removed the co-segregating markers (similarity index = 1.00), which are supposed to be mapped at the same locus on the linkage group. The genetic linkage map was constructed in the Microsoft Excel add-on program, MapDisto v2.1.7 [65] using the filtered marker data. The “find linkage groups” command was used to identify the linkage groups with default recombination frequency (RF) of 0.3 and log of odds (LOD) values ranging from 3 to 12. The “order sequence” command was used to perform preliminary marker order in the selected linkage group, followed by the “ripple order” command to verify the local orders in the linkage group. The “check inversions” command was used to check and correct the marker order for local inversions, and finally, the “drop locus” command was used to drop one marker at a time if any marker caused an important negative difference in the map size. Kosambi mapping function [66] was used to convert recombination fractions into map distances in centimorgans (cM). Co-segregating “similarity loci” markers, which were removed from the initial linkage analysis, were included in the final map to their respective locus positions. MapChart v2.2 [67] was used to graphically draw the linkage maps.

### 4.6. QTL Mapping

Prior to QTL analysis, all the phenotype data were assessed for normality using the Shapiro–Wilk normality test [38] and transformed by Box–cox transformations [68] using statistical package R v3.4.3 [58]. QTL analysis was performed for each environment separately. A combined QTL analysis was also performed using BLUP extracted from the phenotypic data of each genotype across all environments. Initially, WinQTL Cartographer v2.5 [69] was used to detect QTL in the mapping population. The composite interval mapping (CIM) [70] option of the program was chosen for a QTL scan across the sunflower genome using the standard model (model 6) with forward and backward regression method. The program was optimized to select up to five control markers with a window size of 10 cM and a walk speed of 1 cM. Genome-wide significance LOD threshold values for each environment were determined independently using 1000 times permutation tests [71]. The results of the WinQTL Cartographer were verified by running a QTL analysis with the same datasets in other software developed using the same or different algorithms and/or options, including the QGene v4.3 software [72], PLABQTL software v1.2 [73], R/qtl package v1.44-9 [74], and QTL IciMapping v4.1 software [75]. We compared the analyses output of all programs and reported only those QTL in this study that were detected with significant LOD values in at least two programs within the same genomic regions. A 95% confidence interval was used to estimate the left and right margins of the QTL using 1-LOD of the most likely QTL peak position. The linkage map and QTL positions were drawn using the software MapChart 2.2 [67]. For naming of the PSC resistance QTL in sunflowers, we followed the convention proposed by Talukder et al. [46], where the name of the QTL started with a prefix Q, followed by a three-letter descriptor of the phenotype, the LG number, and a serial number.

## Figures and Tables

**Table 1:** Combined analysis of variance (ANOVA) for Phomopsis stem canker disease incidence scores among HA 89/HA-R3 recombinant inbred lines tested in multiple environments during 2016 to 2018.

Component	df	Variance Estimate	Confidence Limit (0.05)		*F*/*Z* Value †	*p*-Value > *F*/*Z*
			Lower	Upper		
Genotype (Gen)	165	-	-	-	**4.23**	**<0.0001**
Environment (Env)	6	$\sigma_{l}^{2}\;$= 213.35	87.14	1091.70	1.69	0.0454
Rep (Env)	14	$\sigma_{r}^{2}\;$= 11.48	5.73	33.50	2.32	0.0101
Gen × Env	987	$\sigma_{gl}^{2}\;$= 136.96	118.57	160.00	13.10	<0.0001
Error	2303	$\sigma_{e}^{2\;}$= 260.59	246.10	276.42	33.74	<0.0001

† In the PROC MIXED model, genotypes were considered fixed and, therefore, subject to *F*-test (values in bold). *F*, Fisher’s *F*-test statistic; *Z*, *Z*-test statistic.

**Table 2:** Spearman rank correlations (ρ) between Phomopsis stem canker disease incidence scores in the HA 89/HA-R3 recombinant inbred line population tested in multiple environments during 2016 to 2018.

Environment	Crookston 2016	Rothsay 2016	Grandin 2016	Rothsay 2017	Crookston 2017	Glyndon 2017
Crookston 2016	-	-	-	-	-	-
Rothsay 2016	0.42 ***	-	-	-	-	-
Grandin 2016	0.31 ***	0.50 ***	-	-	-	-
Rothsay 2017	−0.08	0.15	0.35 ***	-	-	-
Crookston 2017	0.22 **	0.38 ***	0.50 ***	0.50 ***	-	-
Glyndon 2017	0.15	0.39 ***	0.51 ***	0.43 ***	0.57 ***	-
Staples 2018	0.27 ***	0.38 ***	0.59 ***	0.16 *	0.37 ***	0.43 ***

* Significant at the 0.05 probability level, ** Significant at the 0.01 probability level, *** Significant at the 0.001 probability level.

**Table 3:** Summary of sunflower linkage map developed using single nucleotide polymorphism (SNP) markers in the HA 89/HA-R3 recombinant inbred line population.

Linkage Group	Map Length (cM)	No. of Loci	No. of Markers	cM/Locus	cM/Marker
LG1	112.30	85	107	1.32	1.05
LG2	126.68	118	145	1.07	0.87
LG3	42.18	52	82	0.81	0.51
LG4	107.78	104	156	1.04	0.69
LG5	119.56	78	92	1.53	1.30
LG6	40.43	48	62	0.84	0.65
LG7	46.03	39	60	1.18	0.77
LG8	130.90	146	206	0.90	0.64
LG9	68.99	60	97	1.15	0.71
LG10	70.20	59	85	1.19	0.83
LG11	71.63	94	125	0.76	0.57
LG12	60.01	47	61	1.28	0.98
LG13	109.19	130	164	0.84	0.67
LG14	77.06	86	113	0.90	0.68
LG15	56.61	57	82	0.99	0.69
LG16	133.38	63	78	2.12	1.71
LG17	132.41	127	164	1.04	0.81
Total	1505.34	1393	1879	1.08	0.80

**Table 4:** Quantitative trait loci for Phomopsis stem canker resistance identified in combined analysis using best linear unbiased predictor (BLUP) of integrated disease incidence data collected from the HA 89/HA-R3 recombinant inbred line population across seven environments during 2016 to 2018.

QTL	LG	Pos (cM)	LOD	Left Marker	Pos (cM)	Right Marker	Pos (cM)	*R* ^2^	Additive	Resistance Allele
*Qpsc*-2.1	2	90.00	7.04	S2_84829945	88.69	S2_99451880	91.45	6.89	3.70	HA 89
*Qpsc-3.1*	3	13.47	3.88	S3_22684279	13.33	C3_24497005	13.61	14.10	5.56	HA-R3
*Qpsc-3.2*	3	20.00	6.20	S3_59514629	16.80	C3_112733070	20.22	11.80	6.23	HA-R3
*Qpsc-4.1*	4	76.00	8.52	S4_204504697	75.46	S4_187776941	78.62	5.28	4.73	HA 89
*Qpsc-5.1*	5	79.50	4.34	C5_57562566	77.31	S5_35209351	79.91	6.53	3.02	HA-R3
*Qpsc-5.2*	5	86.19	6.19	S5_97363446	84.80	C5_95220778	86.78	7.95	5.22	HA-R3
*Qpsc-8.1*	8	49.10	8.78	S8_19807421	42.75	S8_21996456	49.40	11.79	4.10	HA-R3
*Qpsc-10.1*	10	22.00	7.54	C10_101030161	21.71	S10_8272308	26.65	10.50	4.39	HA 89
*Qpsc-11.1*	11	35.40	6.50	S11_41445957	33.95	S11_44270406	35.57	12.32	4.37	HA 89
*Qpsc-12.1*	12	25.00	5.62	S12_38476421	19.47	C12_63578064	26.01	9.10	3.27	HA-R3
*Qpsc-13.1*	13	90.60	6.87	S13_163494737	90.33	S13_158935119	90.60	13.60	3.45	HA-R3
*Qpsc-13.2*	13	99.00	7.96	C13_161608693	97.77	C13_180087451	99.03	5.24	4.90	HA-R3
*Qpsc-16.1*	16	10.00	3.62	S12_172320483	5.69	S16_195063009	10.92	10.90	4.89	HA-R3
*Qpsc-17.1*	17	29.00	13.31	S14_47547220	24.81	S13_165042726	29.45	9.76	5.66	HA 89
*Qpsc-17.2*	17	40.00	6.13	S17_170827390	39.82	C17_170617819	41.21	17.39	10.09	HA-R3

QTL—quantitative trait loci; LG—linkage group; LOD—log of odds; Pos—position; ***R*^2^**—phenotypic variation explained.

## Supplementary Materials

The following are available online at https://www.mdpi.com/1422-0067/21/4/1497/s1. Figure S1: Heat map showing Phomopsis stem canker disease incidence of the parents and 164 recombinant inbred lines of the HA 89/HA-R3 mapping population evaluated across seven environments of North Dakota and Minnesota during 2016 to 2018. Table S1: Detail of sunflower linkage map developed using SNP/InDel markers in the HA 89/HA-R3 recombinant inbred line population. Table S2: Numbers of distorted SNP/InDel markers and markers from other chromosomes. Table S3: QTL associated with Phomopsis stem canker resistance identified in both combined and individual environment analysis in HA 89/HA-R3 recombinant inbred line population tested in multiple environments during 2016 to 2018. Table S4: Flanking sequence of SNP/InDel markers associated with Phomopsis stem canker resistant QTL identified in HA 89/HA-R3 sunflower recombinant inbred line population.
