# Supplementary figures and images for: Genetic Dissection of Phomopsis Stem Canker Resistance in Cultivated Sunflower Using High Density SNP Linkage Map
Source: Int J Mol Sci. 2020 Feb 22;21(4):1497. doi: 10.3390/ijms21041497 (PMC7073018; doi:10.3390/ijms21041497)

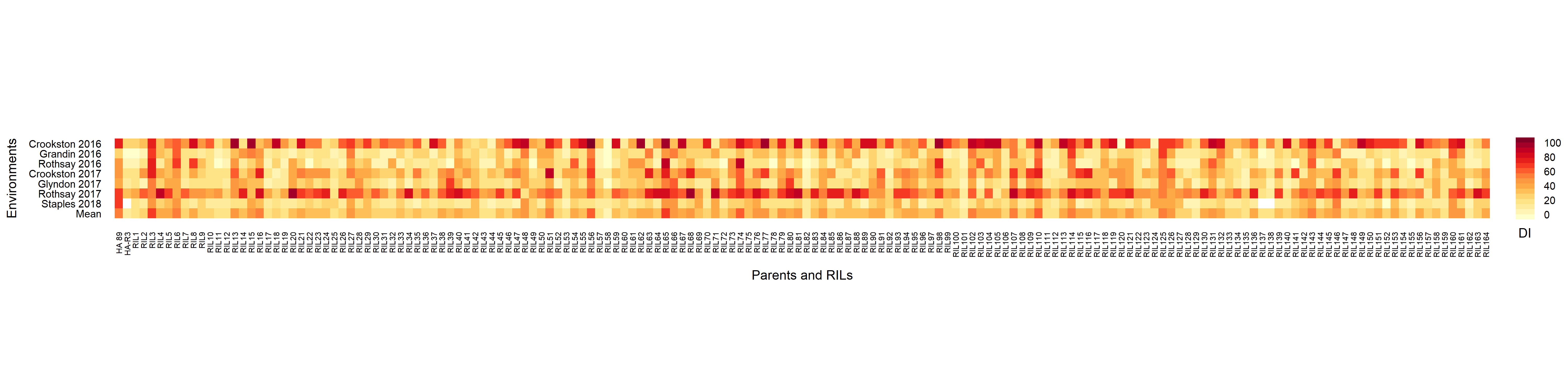

Supplement: Supplementary file 1 [file ijms-21-01497-s001.zip › Supplemental files/Figure S1.tiff]
